# Psychotic Symptoms as the Initial Presentation of a Long-Lasting Misdiagnosed Anti-GAD65 Autoimmune Encephalitis: An Emblematic Case and Literature Review

**DOI:** 10.3389/fpsyt.2022.754938

**Published:** 2022-02-23

**Authors:** Jianjun Wang, Shenglan Gong, Fanxin Kong, Dongbin Cai, Binqing Huang, Haotao Zheng, Songjun Lin, Jinfang Li, Tianfeng Zhang

**Affiliations:** ^1^Fourth Clinical Medical College, Guangzhou University of Chinese Medicine, Shenzhen, China; ^2^Department of Neurology and Psychology, Shenzhen Traditional Chinese Medicine Hospital, Shenzhen, China; ^3^Global Clinical Scholars Research Training (GCSRT), Harvard Medical School, Boston, MA, United States; ^4^Shenzhen Hospital (Futian) of Guangzhou University of Chinese Medicine, Shenzhen, China; ^5^Sixth Clinical Medical College, Guangzhou University of Chinese Medicine, Shenzhen, China

**Keywords:** autoimmune, encephalitis, psychosis, GAD65, autoimmune hepatitis, autoantibodies, seizure (medical records)

## Abstract

**Objective:**

To present a long-lasting misdiagnosed case of anti-GAD65 autoimmune encephalitis (AE) and promote the early identification of reversible psychotic symptoms in AE.

**Methods:**

The case report was generated through detailed assessment of clinical characteristics, cerebral magnetic resonance images, and laboratory results. Meanwhile, a literatures review related to the topic was conducted.

**Results:**

Psychotic symptoms could be presented in the early stage of anti-GAD65 autoimmune encephalitis. Even though there exists a transdisciplinary gap that hinder the timely recognition of early psychiatric symptoms as components of organic disease, a few strategies could be introduced to enable the earlier recognition and appropriate treatment.

**Conclusions:**

Our report intends to raise awareness to promote the early identification of immune-mediated “symptomatic” forms of psychosis.

## Introduction

Autoimmune encephalitis (AE) is a rare, debilitating and potentially fatal immune-mediated disease characterized by seizures, cognitive impairment, and psychiatric symptoms (1, 2). The psychiatric symptoms were prominent when AE was recognized more than a decade ago (3). Mane-Damas et al. claimed that patients with AE showed marked psychotic symptoms prior to overt neurological manifestations and were admitted to a psychiatric hospital (4, 5). In addition, the psychotic manifestations are similar to those observed in primary functional psychiatric disorders, making misdiagnosis or underdiagnosis occur in AE patients with psychotic symptoms.

Glutamic acid decarboxylase 65 (GAD65) antibody is a subtype of new intracellular antigens and associated with limbic encephalitis (LE) (6, 7). As core manifestations of LE, cognition impairments and seizures have been well-described in patients with GAD65 encephalitis (6–8), whereas psychiatric symptoms remain an open topic. Since GAD was hypothesized as the rate-limiting enzyme in the synthesis of γ-aminobutyric acid (GABA), and dysfunctions of GABAergic neurons were associated with the pathogenesis of psychiatric manifestations (6, 9, 10), it is worthwhile to explore the psychiatric manifestations that would complicate the diagnosis of GAD65 encephalitis.

In this case report, we reported a Chinese female who initially showed episodic psychotic symptoms and was finally diagnosed as GAD65 encephalitis. The comprehensive neurological and psychiatric characteristics were assessed and the potential clues for early diagnosis were emphasized. The informed consent was obtained from the patient. Furthermore, a literature review related to the topic was conducted.

## Case Description

Mrs. K is a 30-year-old female, who initially suffered from insomnia, auditory hallucinations, and behavioral abnormalities (e.g., grabbing food with hands) 2 years ago. These symptoms were initially sporadic and gradually progressed with episodes of generalized tonic-clonic seizures, which lasted for 3 to 5 min and occurred 1 to 2 times a month. According to her chart, two past admissions for similar recurrent seizures and behavior deficits were recorded in two general hospitals. The electroencephalogram (EEG) results demonstrated that the rhythmic epileptiform discharges arose out of the bilateral temporal regions with right predominance. The thyroid hormones, folate, B12 vitamin and serum sodium concentrations were normal. The routine cerebrospinal fluid (CSF) analysis (including oligoclonal bands) and brain magnetic resonance imaging (MRI) scan without contrast were unremarkable. Therefore, she was diagnosed with epilepsy (ICD-10 Code, G 40) and suspicious encephalitis (ICD-10 Code, G 04.9) in the two previous hospitals, respectively. Sodium valproate (1,000 mg/d), carbamazepine (200 mg/d), and olanzapine (2.5 mg/d) were prescribed and taken. However, the seizures were only partially alleviated, and obvious behavior abnormalities were progressed in social settings. Moreover, her husband reported that she had paranoid insecurity (e.g., crying or not sleeping) and a quality of imperative auditory hallucination (e.g., kneeling on the road for ancestor worship). Then, she was admitted to a psychiatric hospital, where her symptoms were not successfully controlled with sodium valproate (1,500 mg/d), carbamazepine (600 mg/d), topiramate (200 mg/d), and risperidone (2 mg/d). Although the frequency of chaotic behaviors and seizures were decreased, she became confused, minimally responsive to directions and communications, and even could not recognize her family members.

**Figure 1:**
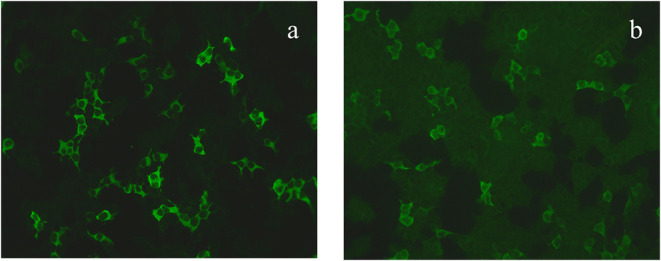
The GAD65 antibody was diluted 1:10 in the serum **(a)** and 1:100 in the cerebrospinal fluid **(b)**.

**Figure 2:**
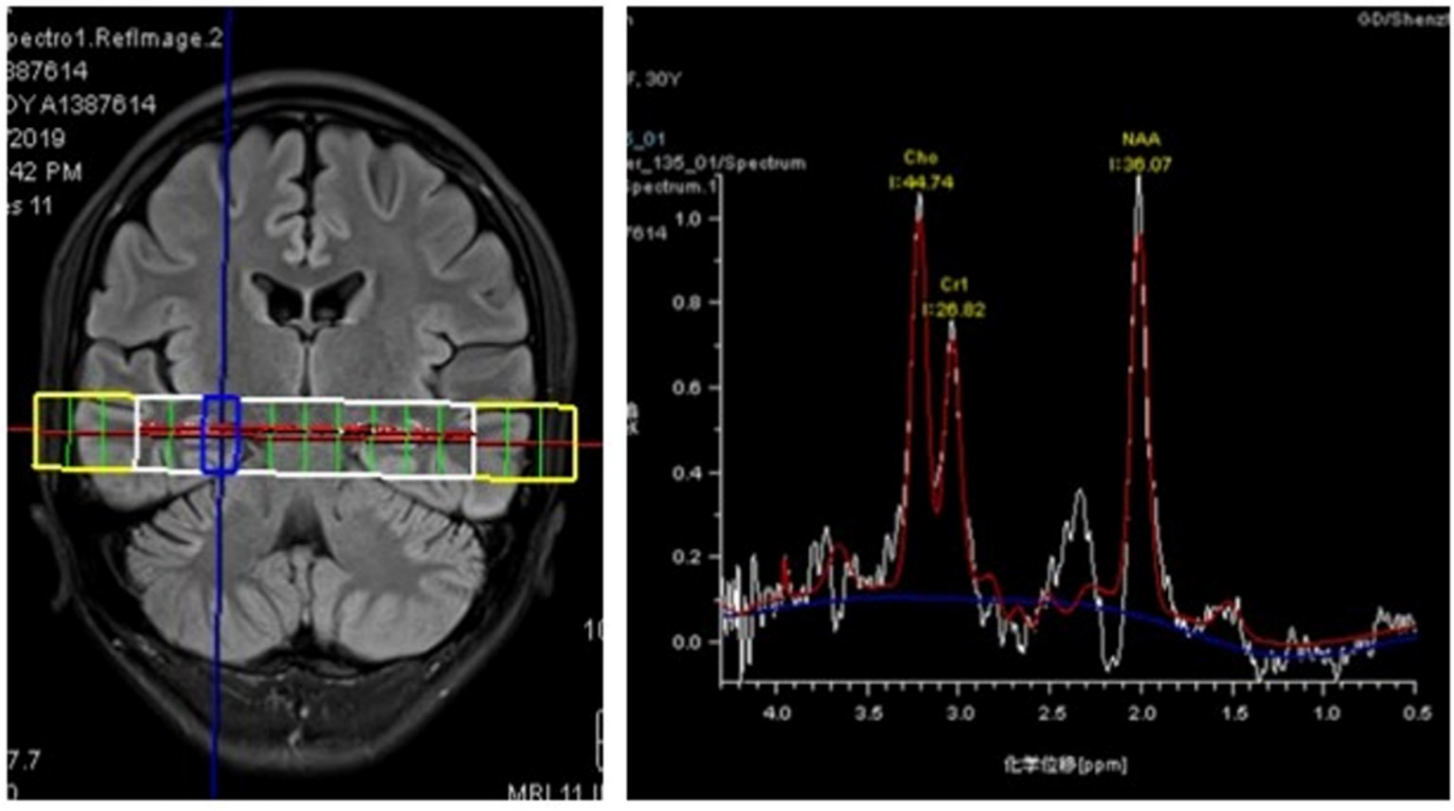
^1^H-magnetic resonance spectroscopy (MRS) images showing an increased Cho/Cr ratio (1.67) in the right hippocampus with a markedly elevated Cho peak.

The patient was then admitted to our facility in September 2019. The general physical and neurological examinations were unremarkable. The mental status examination revealed blunted effect, slow response, inappropriate laughing or crying, and episodes of auditory hallucination from her dead mother (e.g., telling her to dash into traffic, or that her son got lost). She could not realize these disorganized behaviors until she got relieved by the following treatments. Consistent to previous EEG findings, rhythmic epileptiform discharges were confirmed. Further, a thorough evaluation for organic causes was performed, including brain MRI with contrast, lumbar puncture (LP), an autoimmune workup and positron emission tomography-computed tomography (PET/CT). The serum and CSF antibodies to eight neuronal proteins were assessed, including NMDA-R, GAD65, a-amino-3-hydroxy-5-methyl-4-isoxazole prophetic acid receptor 1/2 (AMPAR1/2), gamma-aminobutyric acid B receptor (GABA B), leucine-rich glioma inactivated 1 (LGI1) and contacting-associated protein-like 2 (CASPR2), dipeptidyl peptidase protein-like 6 (DPPX), and immunoglobulin-like cell adhesion molecule 5 (IgLON5). The anti-GAD65 antibody titer was positive in CSF (++ 1:10) and in serum (++ 1:100) (Figures 1a,b). Blood and CSF tests for viral or bacterial infections were negative. It was interesting that a rapid improvement of verbal response and psychiatric symptoms were repeatedly observed after LP. Due to the association of anti-GAD65 autoantibodies with tumors, a whole-body 18F-fludeoxyglucose PET/CT was further performed, revealing no signs of tumor. The paraneoplastic antibody detection in CSF was also unremarkable. Conventional structural MRI was negative. However, ^1^H-magnetic resonance spectroscopy (MRS) revealed a markedly elevated Cho peak, thus leading to an increased choline (Cho)–to-creatine (Cr) ratio (Cho/Cr: 1.67) in the right hippocampus, compared with the left mirrored hippocampus (Figure 2).

Based on the clinical presentation, EEG and laboratory findings, a GAD65-mediatd autoimmune encephalitis (ICD-10 Code, G 04.9) was confirmed. The patient was treated first with a 5-day course of intravenous immunoglobulin (0.4 g/kg) and 3-day course of methylprednisolone (1,000 mg/day), followed by an oral prednisolone therapy (started at a dose of 50 mg/day). The immunosuppressive treatment resulted in a moderate improvement of behavior disturbance and seizures until symptoms deteriorated after months. The patient was then treated with another two cycles of intravenous immunoglobulin and methylprednisolone in the following sessions. Moreover, a combination of methylprednisolone (1,000 mg/day for 3 consecutive days) and monthly intravenous pulses of cyclophosphamide (500 mg/m^2^ body surface) was introduced in the latest session. We observed moderate remission after half a year follow-up. She was free of seizures, positive psychotic symptoms, or disorganized behavior with the augmented oral anticonvulsant and antipsychotics (topiramate 125 mg/d, sodium valproate 1,500 mg/d, and olanzapine 2.5 mg/d). Although she experienced frequent insomnia, slow response, and blunted effect, preventing her from working properly, she could stay at home alone and take care of herself. The score on the modified Rankin scale was reduced from 4 to 2.

## Discussion

Although this patient presented typical features of LE (11), unremarkable findings from routine MRI scan, behavior disturbance, and acute psychotic episodes contributed to the delayed diagnosis. The patient presented psychiatric symptoms as the initial disturbance and responded poorly to antipsychotics, which were not well-recognized in two recent observational studies on GAD65^+^ encephalitis (7, 8). Our case could be served as supplemental evidence to strengthen the early recognition of anti-GAD65 Abs-mediated AE, and to avoid the long-lasting misdiagnosis and delayed immunosuppressive treatment.

LE is usually characterized by short-term memory deficits, seizures, and psychiatric symptoms suggestive of limbic involvement (11). GAD65 Abs has drawn increasing attention as a subtype of intracellular antigens targeting at the limbic system. There have been case series reporting psychotic symptoms related to GAD65 encephalitis (12, 13). However, the evidence about the clinical entity could be controversial. A recent meta-analysis reported a pooled prevalence rate of 5.8% for GAD65 Abs in patients with psychosis (14), which suggested that the psychiatric symptoms are important phenotypes associated with GAD65 Abs and are worthy of attention. In contrast, the psychiatric symptoms were not well-recognized in two recent observational studies on GAD65^+^ encephalitis (7, 8), one of which reported that 14.3% participants (*n* = 5) had behavioral disturbances and stated the deficits of non-standard neuropsychological assessments. Hence, there are increasing debates regarding the comprehensive neuropsychiatric evaluations of psychiatric symptoms in AE (15). On one hand, patients are sometimes first hospitalized in a psychiatric institution as AE can resemble acute psychosis (2), causing concern about the misdiagnosis of AE patients with acute psychosis (16). On the other hand, as advocated, psychiatrists should be involved in the diagnosis and treatment of AE patients, since the scarcity of psychiatric investigations could lead to underdiagnosis of psychiatric phenotypes (15). Therefore, regarding the interpretations of the currently available data on the neuropsychiatric features of AE, including anti-GAD65 patients, we should keep in mind the potential misdiagnosis or underdiagnosis in many instances (2, 6).

Due to the transdisciplinary gap between the neurology and psychiatry, it could be challenging at times to recognize the early psychiatric symptoms as part of the organic disease. It has been reported that the mean time between the occurrence of first symptoms and antibody testing was often alarmingly prolonged from 74 days to 483 days (17). The duration in our case was delayed for a period of 2 years. Thus, there is a great need to raise awareness of early diagnosis of AE for both neurologist and psychiatrist. A few strategies could be introduced. On one hand, the hallucinations in this patient were not systematic but with episodic characteristics, which is clearly different from the persistent psychotic symptoms in primary psychotic disorder as schizophrenia. On the other hand, the patient had multiple red flags for the suspicion of autoimmune encephalitis (17, 18), including seizures, cognitive impairments, insufficient response to antipsychotics, and rapid progression. Additionally, early diagnosis is also urged due to bad outcomes of the delayed immunosuppression treatment. It has been reported that delays in immune therapy administration could lead to treatment resistance and permanent sequelae (19). In our case, the patient received most types of immunotherapies used for AE, including steroids, IVIg, and cyclophosphamide. The treatment seemingly only had moderate effects on the patient's clinical condition. It has been proposed that acute and reversible immune activation in the initial stage of disease could result in significantly better outcomes even without any permanent brain damage (20, 21). Thus, early recognition is mandatory for both AE and newly proposed autoimmune psychosis (18).

Owing to the newly-recognized autoimmune encephalitis mediated by GAD65 Abs, a few interesting phenomena are deserved to be focused on. Firstly, an unexplained clinical improvement after LP was repeatedly observed. Considering the T-cell-based cytotoxicity and the questionable pathogenic significance with intracellular antigens (e.g., GAD65) (22), removing antibody-positive CSF will not have any effects on the antibodies presented in the brain parenchyma. A recent study also reported limited therapeutic effects of immunoadsorption therapy in patients with GAD65 encephalitis (23). It might be infeasible to monitor the antibody titers, even though a decrease of anti-GAD65 concentration was occasionally reported accompanying the clinical improvement (7). Besides, the patient could hardly benefit from the reduced CSF pressure after LP due to the absence of increased CSF pressure or hydrocephalus. Thus, other factors should be fully considered and inspected, such as the frequent presence of coexisting autoantibodies (13, 24). Secondly, an increased Cho peak in the right hippocampus was observed in MRS, which suggests cell destruction or high cellularity (25). Consistently, the corresponding rhythmic epileptiform discharges in right hippocampus and the clinical presentation targeting at the limbic area were also observed. Hence, the MRS could be served as an alternative method to observe microstructure alterations in the absence of routine structure MR findings. Meanwhile, considering the characteristics of MRS in glioblastoma (GBM), it could be further used to differentiate GBM from AE with visible neuroimaging findings in conventional MR scan (26). At last, with the increasing applications of multimodal neuroimaging (e.g., diffusion tensor imaging etc.) in autoimmune encephalitis (27, 28), a better understanding of the specific pathophysiologic properties of encephalitis is warranted, which would be beneficial to promote the early diagnosis of AE.

Taken together, psychotic symptoms could be presented as a component of GAD65-mediated autoimmune encephalitis, and may lead to misdiagnosis and delayed immunosuppressive treatment. Given the interdisciplinary gap in the literature, it is necessary for neurologists and psychiatrists to be aware of the early identification of immune-mediated “symptomatic” forms of psychosis.

## Data Availability Statement

The original contributions presented in the study are included in the article, further inquiries can be directed to the corresponding authors.

## Ethics Statement

The studies involving human participants were reviewed and approved by Ethical Committee of Shenzhen Traditional Chinese Medicine Hospital. The patients/participants provided their written informed consent to participate in this study. Written informed consent was obtained from the individual(s) for the publication of any potentially identifiable images or data included in this article.

## Author Contributions

JW, SG, JL, and FK observed the patient and analyzed the data. JW, SG, DC, and HZ reviewed the literature. BH, SL, FK, JL, and TZ formulated the conception of the study. JW, SG, and FK wrote the paper. All authors approved the final work.

## Funding

This work was supported by National Natural Science Foundation of China (82004284), Guangdong Medical Science Foundation (A2020370 and A2021199), Guangdong Administration of Traditional Chinese Medicine Project (20201419), Shenzhen Science and Technology Research Program (RCBS20200714114959156), and Shenzhen Municipal Health and Family Planning System Scientific Research Project (SZFZ2018013).

## Conflict of Interest

The authors declare that the research was conducted in the absence of any commercial or financial relationships that could be construed as a potential conflict of interest.

## Publisher's Note

All claims expressed in this article are solely those of the authors and do not necessarily represent those of their affiliated organizations, or those of the publisher, the editors and the reviewers. Any product that may be evaluated in this article, or claim that may be made by its manufacturer, is not guaranteed or endorsed by the publisher.
